# Recent advances on fluorescent biomarkers of near-infrared quantum dots for *in vitro* and *in vivo* imaging

**DOI:** 10.1080/14686996.2019.1590731

**Published:** 2019-04-15

**Authors:** Shanmugavel Chinnathambi, Naoto Shirahata

**Affiliations:** a International Center for Young Scientists, National Institute for Materials Science (NIMS), Tsukuba, Japan; b International Center for Materials Nanoarchitectonics, NIMS, Tsukuba, Japan; c Graduate School of Chemical Sciences and Engineering, Hokkaido University, Sapporo, Japan; d Department of Physics, Chuo University, Tokyo, Japan

**Keywords:** Quantum dots, bio-marker, NIR imaging, review, 60 New topics / Others: Nanocrystals, Quantum dots, Bioimaging, Biomarkers, Fluorescence imaging, 204 Optics / Optical applications

## Abstract

Luminescence probe has been broadly used for bio-imaging applications. Among them, near-infrared (NIR) quantum dots (QDs) are more attractive due to minimal tissue absorbance and larger penetration depth. Above said reasons allowed whole animal imaging without slice scan or dissection. This review describes *in vitro* and *in vivo* imaging of NIR QDs in the regions of 650–900 nm (NIR-I) and 1000–1450 nm (NIR-II). Also, we summarize the recent progress in bio-imaging and discuss the future trends of NIR QDs including group II-VI, IV-VI, I-VI, I-III-VI, III-V, and IV semiconductors.

## Graphical Abstract

**Figure:**
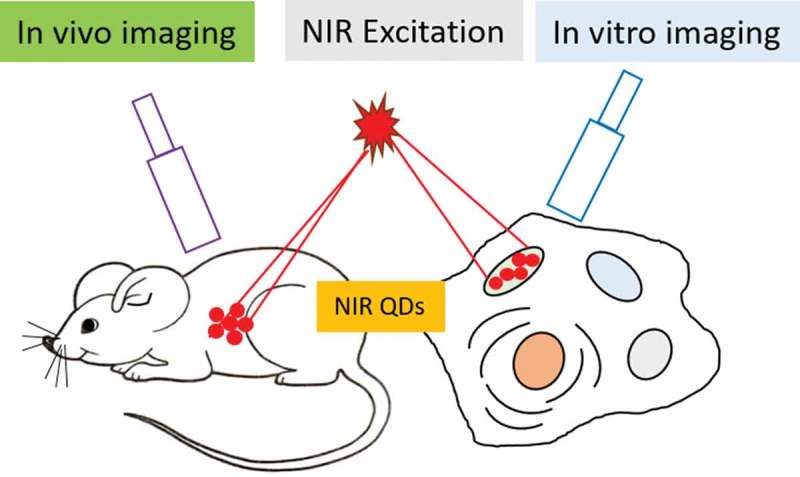

## 1. Introduction

Fluorescence imaging is a useful technique for biological applications. Recently, quantum dots (QDs) have become a popular imaging agent, competing with other fluorescent nanomaterials and dyes [1–7]. One more advantage is we can get multiplex emission with single light source excitation with minimal spectral overlap. The outstanding optoelectronic properties of QDs include high quantum yield (QY), size-dependent, and narrow light emission, broad absorption spectrum, massive stoke shift and resistance to photobleaching [8]. In the blood protein and other bio-molecule, sizes are comparable with most available QDs. QDs are prepared in organic or aqueous solutions depending on the proposed applications. Recently, exciting research activities going on in the above said reasons and quality of the materials. Biological imaging started using QDs from 1998, from that moment QD-based bio-imaging and bio-sensing applications developed rapidly [9–13].

**Figure 1.**
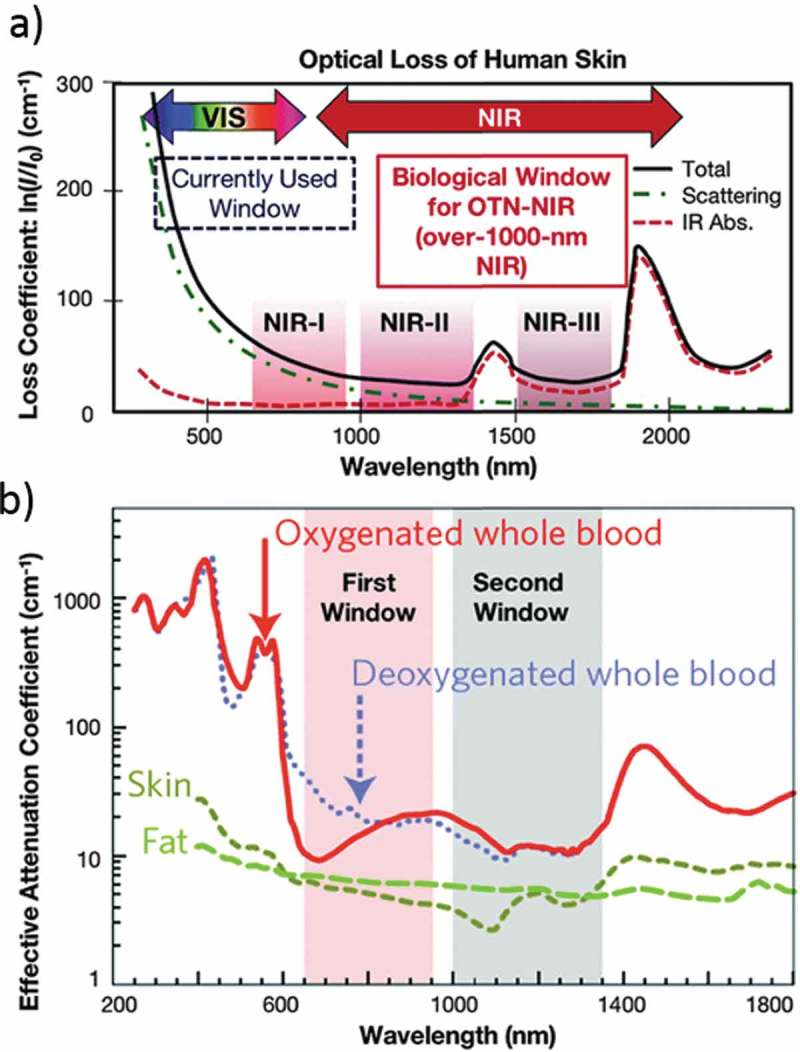
(a) Human skin absorption spectra in the of NIR-I, NIR-II, and NIR-III regions. (b) Absorption and scattering spectra from oxygenated blood, deoxygenated blood, skin and fatty tissue. Reproduced with permission from (Figure 1(a), [14] Copyright 2009 RSC Publishing; Figure 1(b), [18] Copyright 2016 RSC Publishing).

For bio-medical applications, it is better to use near-infrared (NIR) region due to low absorption (see Figure 1(a,b)) and autofluorescence from the tissues [14]. Human tissues are made up of several organic molecules that naturally absorb (DNA, collagen, elastin, proteins, NADH, and FAD) and emit (NADH, FAD, proteins and DNA) the light in the ultraviolet to visible region [9]. The NIR emitting QDs for *in vivo* deep-tissue imaging are desirable owing to the improved tissue penetration of lights and decreased tissue autofluorescence in the NIR range of 750–940 nm and 1200–1700 nm. Hence, researchers are developing fluorescent materials with both the excitation and emission wavelengths confined within the biological transparency window (NIR-I: λ_em_ = 750–900 nm) [15]. NIR-I window reduces the scattering and absorbance (due to H_2_O, boric acid, metabolic acid, fat, bilirubin, melanosome) of the light and resulting deep penetration in biological tissues. Therefore, it is important to select the optical transmission window of the skin (750–940 nm) for NIR imaging. NIR-II window (λ_em_ = 1000–1700 nm) was developed for imaging with a tissue penetration depth of more than 2 cm.

**Figure 2.**
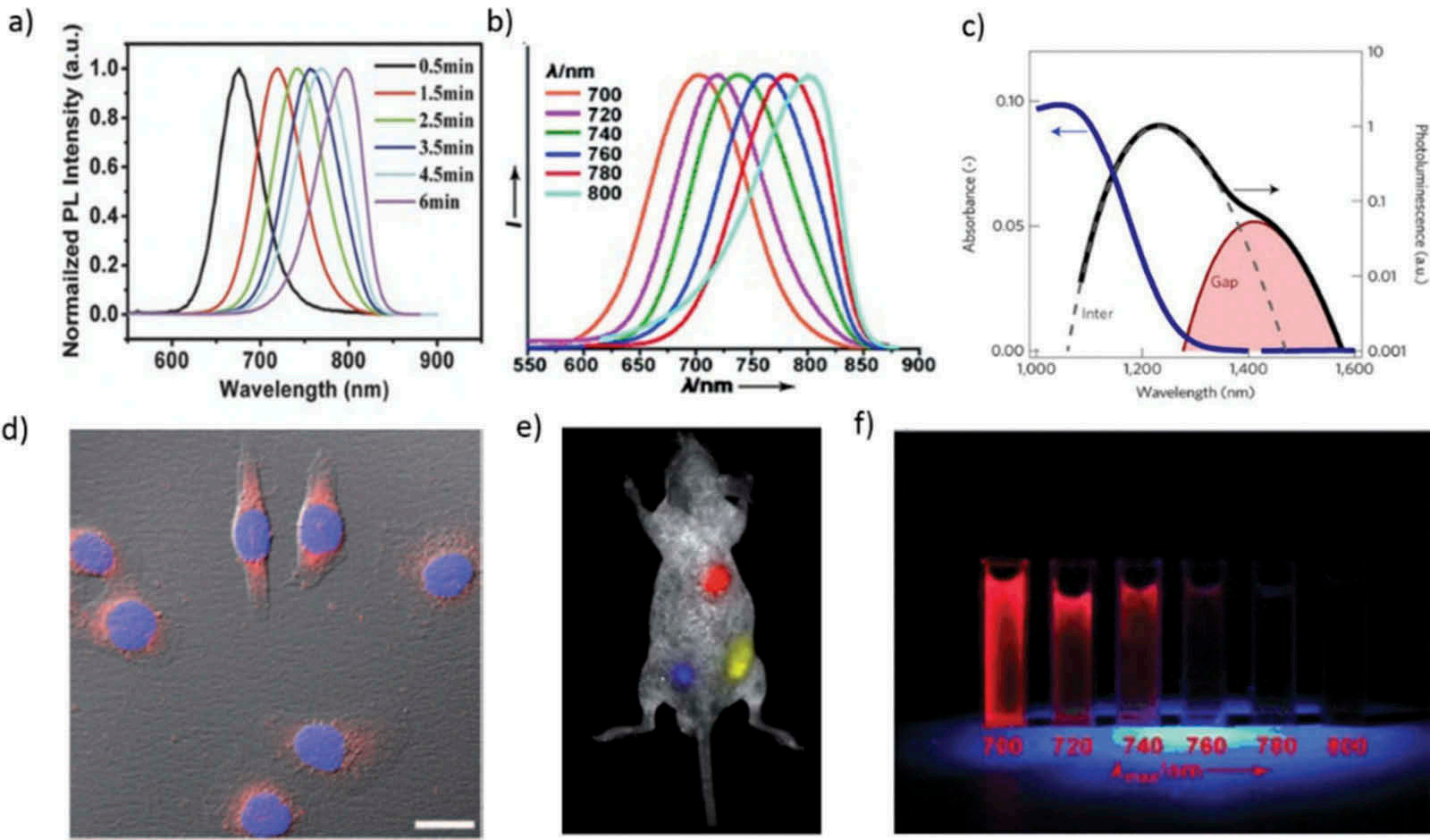
(a) PL emission spectra of CdTe_0.15_ Se_0.85_ QDs under different growth times. (b) NIR QDs with emission tunable from 700–800 nm (excitation = 450 nm). (c) Absorbance (blue) and emission (black) of HgTe QDs suspended in TCE. (d) Dual-color immunofluorescence cellular imaging (scale bar: 20 mm). The Hela cells are labeled by the QDs/protein conjugates (red) and Hoechst (blue). (e) Multiplex imaging capability of CdTe/CdSe QDs in live animals. f) CdTe QDs under UV irradiation (λ_ex_ = 365 nm). Reproduced with permission from (Figure 2(a), [21] Copyright 2014 Wiley Publishing; Figure 2(b), [22] Copyright 2011 Wiley Publishing; Figure 2(c), [23] Copyright 2018 Nature Publishing Group; Figure 2(d), [22] Copyright 2011 Wiley Publishing; Figure 2(e), [29] Copyright 2011 RSC Publishing; and Figure 2(f), [22] Copyright 2011 Wiley Publishing).

Most importantly, QDs must be dispersed in water for biological applications. QDs are synthesized initially in organic nonpolar solvents then surface modification essential to soluble in water. For bio-imaging, bio-sensing, researchers developed NIR QDs with various surface functionalization to get the water-soluble materials [16–19]. The large QDs surface area gives better attachment of the bio-molecules compared with other organic dyes and other bio-molecules. The conventional methods to prepare the water-dispersible QDs are ligand exchange and surface modification with some hydrophilic molecules [20]. Most importantly, QDs are more stable than organic dyes upon light exposure. The main content of this review is QDs which can be emitted in NIR-I and NIR-II regions. Here, mainly we presented the properties of NIR-emitting QDs for bio-imaging applications. Table 1 shows the types of NIR QDs, surface modifications, PL properties, and biological applications. We systematically summarize the recent progress of bio-medical imaging and discuss the shortcomings, challenges, and opportunities in it.

**Table 1.** Summary of representative QDs and their surface modifications, emission maxima and bio-applications.

Types of QDs	Surface coating	Emission Maximum (nm)	Applications	Ref
II-VI Group				
CdTe	mercaptoundecanoic acid (MPA)	700–800	*In vivo* tumor targeting	[22]
CdTe/CdSe	PMAS-PEG	801	Multiplex imaging in live animals	[29]
CdTe/ZnSe	phospholipids	730	Imaging of panc-1 tumor-bearing mice	[28]
CdTe1−xSex/CdS	PEG-grafted phospholipid	845	Cancer imaging and therapy	[24]
CdHgTe/CdS/ZnS	N-acetyl-L-cysteine	650–800	*In vivo* tumor targeting	[26]
IV-VI Group				
PbS PbS PbS PbS	recombinant protein (GST-EGFP-GB1)	1150	Breast tumor imaging	[44]
	dihydrolipoic acid – polyethylene glycol	1300	Mice *in vivo*	[45]
	MPA	1100	Imaging of the thrombotic state in septic mouse brain	[35]
	BSA-functionalized AuNCs	813	Bio-imaging of ascorbic acid	[38]
PbS/CdS/ZnS	MPA	1270	*In vivo* fluorescent imaging agents	[39]
I-VI Group				
Ag_2_Se Ag_2_Se Ag_2_Se	polyethylene glycol (PEG)	1300	Blood Clearance and bio-distribution	[48]
	Multidentate polymer	950–1250	*in vivo* fluorescence images	[54]
	C_18_-PMH-PEG	1300	*In vivo* imaging of live mice	[58]
Ag_2_S Ag_2_S Ag_2_S	Glutathiose	1015	nitric oxide delivery and fluorescence imaging	[53]
	Dihydrolipoic acid	1000–1400	early stage tumor diagnosis	[60]
	carboxylic acid group	825	EGFR targeted tumor imaging	[72]
I-III-VI Group				
CuInS_2_	RNase A capped CuInS_2_	700–800	*in vivo* imaging	[74]
AgInSe_2_-ZnSe	multidentate polymer	625–940	Cell imaging	[78]
AgInS_2_	multidentate polymer	820	*in vivo* imaging	[76]
III-V Group				
InAs core-shell	Phospholipid Nanosomes Composite particles	1280 1300 1280	Multicolor imaging Quantitative metabolic imaging Blood flow maps	[84]
InAs/InP/ZnSe	MPA	800	Tumor detection in living mice	[85]
IV Group				
Carbon	Ethylenediamine	750	Two-photon fluorescence imaging	[96]
Carbon	S, Se-codoped	731 & 820	fluorescence imaging and photothermal therapy	[95]
Carbon	Poly(vinylpyrrolidone)	750	*In vivo* NIR fluorescence imaging	[97]
Carbon	PEG-800	600–900	Photothermal Cancer Therapy	[92]
Carbon	Fluorescein isothiocyanate	683	two-photon fluorescence bio-imaging	[93]
Si	Pluronic F127	650	two-photon fluorescence cell imaging	[107]
Si	4,7-di(2-thienyl)-2,1,3-benzothiadiazole	900	Two-Photon Absorbing Antenna	[140]
Si	B,P – codoped	600–1200	time-gated fluorescence imaging in the second near-infrared window	[141]

## 2. Quantum dots for biological imaging

### 2.1. Group II-VI quantum dots

CdTe and HgTe QDs are most widely used in the NIR region. Gao et al. developed CdTe_x_Se_1– x_/ZnS alloy nanocrystals to achieve QYs up to 80% with controllable rod-shape and NIR (*λ*_em_ = 650–870 nm) emission (Figure 2(a)) [21]. He et al. reported a facile one-step microwave synthesis of water-soluble CdTe QDs. The water-soluble NIR CdTe QDs (*λ*_em_ = 700–800 nm) are used first time for *in vivo* tumor targeting and also used for *in vitro* imaging after conjugation with protein molecules (Figure 2(b,d,f)) [22]. Recently, Geiregat et al. demonstrated that mercury telluride (HgTe) QDs exhibit size-tunable emission all over the NIR window at thresholds unmatched by any QDs studied before (Figure 2(c)) [23]. Alloyed CdTe_1−x_Se_x_/CdS NIR QDs are used for detecting pancreatic cancer in mouse models [24]. Alloyed CdHgTe QDs were prepared via heating a mixture of Cd^2+^, Hg^2+^, and Te^2-^ in the presence of 3-mercaptopropionic acid (MPA) as ligands with photoluminescence (PL) QYs (20–50%) and narrow emission bands and it will be a suitable fluorescent probe in the imaging of living animals [25]. Recently, Liu et al. developed a NIR-emitting CdHgTe/CdS/CdZnS QDs and coated them with three different thiol ligands, 3-MPA, thioglycolic acid (TGA), and N-acetyl-L-cysteine (NAC). *In vivo* toxicity measurement shows negligible harmful effects to nude mice even at a concentration of 20 mg kg^−1^ [26]. Gadolinium-functionalized CdHgTe/ZnS core/shell QDs are used for *in vivo* fluorescence and magnetic resonance imaging [27]. Cyclic arginine-glycine-aspartic acid conjugated micelle-encapsulated NIR CdTe/ZnSe QDs as highly luminescent probes for bio-labeling and *in vivo* imaging of pancreatic tumor in live mice [28]. In 2011, CdTe/CdSe QDs were also successfully applied for the fluorescence imaging of living animals (Figure 2(e)) [29].

MPA-stabilized NIR-emitting CdTe/CdS QDs have used fluorescence sensor for Cu^2+^ with a low detection limit, and the sensor was applied to the bio-sensing and bio-imaging in the HeLa cells and Kunming mice. The quenching effects resulted from the aggregation of QDs induced by the competitive binding between MPA on the surfaces of the NIR QDs and the Cu^2+^ present in the solution. The Cu-S bond has a much lower K_sp_ value than that of Cd-S bond. *In vitro* imaging of HeLa cells and *in vivo* imaging of Kunming mice demonstrated that these NIR QDs are suitable for high-resolution imaging of biological tissues owing to the suitable wavelength range of emission light [30].

Lu et al. reported, short-term and long-term *in vivo* bio-distribution, pharmacokinetics, and toxicity of the CdTe QDs. NIR-emitting QDs are initially accumulated in the liver, spleen, and lung for short-time (0.5–4 h) post-injection, and then increasingly absorbed by kidney during long-time (4–94 days) blood circulation. Furthermore, histological and biochemical analyses and body weight measurements demonstrate that there is no overt toxicity of NIR-emitting QDs in mice even during a 94-day exposure [31].

### 2.2 Group IV-VI quantum dots

**Figure 3.**
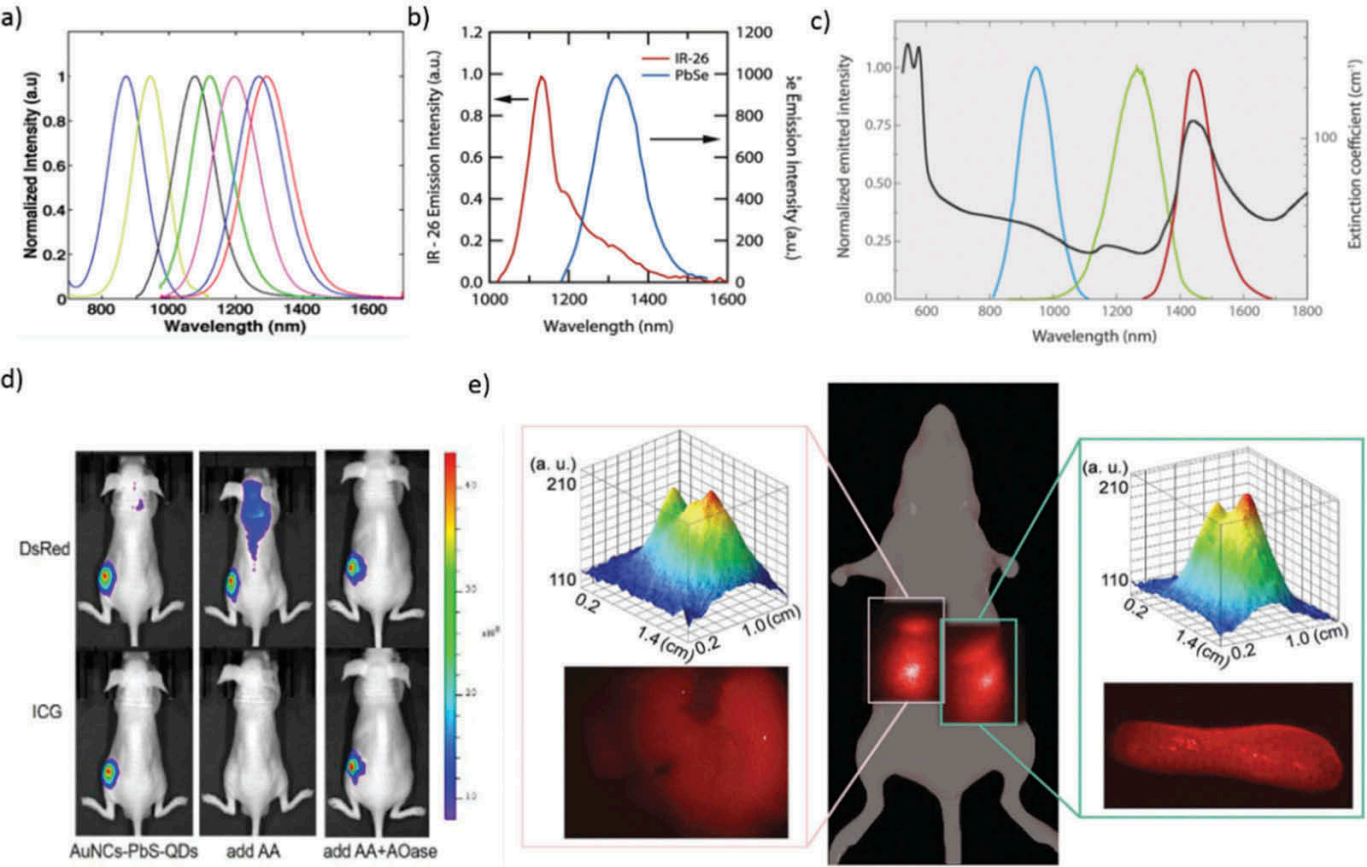
(a) PL spectra of the PbS QDs synthesized by the droplet-based capillary reactor. (b) Comparison of relative PL spectra for IR-26 and a 0.98 eV PbSe QD. (c) The spectral range of different PbS/CdS/ZnS QDs. (d) The nude mice injected with AuNCs-PbS-QDs, AuNCs-PbS-QDs/AA, and AuNCs-PbS-QDs/AA/AOase, respectively. All the images were taken under two filters of DsRed and ICG. (e) Whole body and corresponding intensity surface, and *ex vivo* NIR fluorescence images of the liver (left inset) and spleen (right inset) of a mouse 24 h after tail vein injection of LNH_2_ capped QDs. Reproduced with permission from (Figure 3(a), [33] Copyright 2014 The American Chemical Society; Figure 3(b), [32] Copyright 2010 The American Chemical Society; Figure 3(c), [39] Copyright 2015 Wiley Publishing; Figure 3(d), [38] Copyright 2015 The American Chemical Society; and Figure 3(e), [45] Copyright 2018 RSC Publishing).

Group IV-VI semiconductor QDs are an excellent candidate for biomedical applications due to their unusual electronic and transport properties. QD material purity, surface passivation, reproducible, and high quality were analyzed by a powerful tool like PL characterization. (Figure 3(b)) [32]. Lignos et al. demonstrated a controlled droplet-based microfluidic platform to produce monodisperse PbS and PbSe QDs under a wide range of experimental conditions (Figure 3(a)) [33]. The lowest-energy exciton transitions in PbSe, with diameters between 3 and 8 nm, happen at wavelengths between 1.0 and 1.85 μm [34]. Imamura et al. reported PbS QDs and their use for *in vivo* NIR fluorescence imaging of cerebral venous thrombosis in septic mice [35]. PbS QDs were synthesized using an aqueous route with l-cysteine (l-Cys) as the capping ligands [36]. Tan et al. synthesized NIR-emitting PbSe QDs with controlled size and shape, with tunable emission in the range from 1200 to 1500 nm. The cell viability of mouse fibroblast cells and human liver cancer cells was retained at 80% even in the presence of a silica shell coated QD loading of 100 μg/mL [37]. Nanohybrid Au nanoclusters (NCs) -PbS-QDs was synthesized by the co-template method to obtain the NIR-far red dual emission fluorescence. This material is highly suitable for high-resolution bio-imaging because its emission falls in the ‘optical window’ of *in vivo* imaging with massive Stokes shift and brilliant separation of two emissions (Figure 3(d)) [38]. High-quality, water-dispersible PbS/CdS/ZnS QDs probe lying within the NIR-II window (1000–1350 nm), which leads to higher penetration depths due to the low extinction coefficient of tissues in this spectral range (Figure 3(c)) [39].

Deng et al. developed a facile and environmental-friendly water-soluble PbS QDs with strong fluorescence in the NIR spectral range 870–1010 nm, by using dihydrolipoic acid (DHLA) as the stabilizer [40]. NIR light-emitting luciferase via a bio-mineralization process was reported by Ma et al. The finding that luciferase remains active and enables bio-luminescence resonance energy transfer within the Luc8-PbS complex represents a significant advance over previous templated nanomaterial synthesis [41]. Pietryga et al. reported a novel method which takes advantage of the reactivity of PbSe QD surface to produce PbSe/CdSe core/shell QDs [42]. The water-soluble conjugated structures, consisting of magnetic nanoparticles (NPs) connected to PbSe were useful for biological applications such as bio-sensing, detection of cancer cells, and drug delivery [43]. Breast tumor cellular imaging was performed using GST-EGFP-GB1 protein-coated PbS QDs with a dual-emission band of visible (515 nm) and second-NIR fluorescence (1150 nm) [44]. Zamberlan et al. reported the development of QDs that combine the beneficial bio-compatibility and stability offered by DHLA–PEG-ligands with the tunability of the emission of PbS QDs in the NIR range of low absorption of biological tissues (Figure 3(e)) [45]. Recently, PbS QDs were prepared in sizes between 2.4 and 3.2 nm using PbCl_2_, elemental S, dodecanethiol, and a toluene/oleylamine mixture at low temperatures [46].

### 2.3. Group I-VI quantum dots

**Figure 4.**
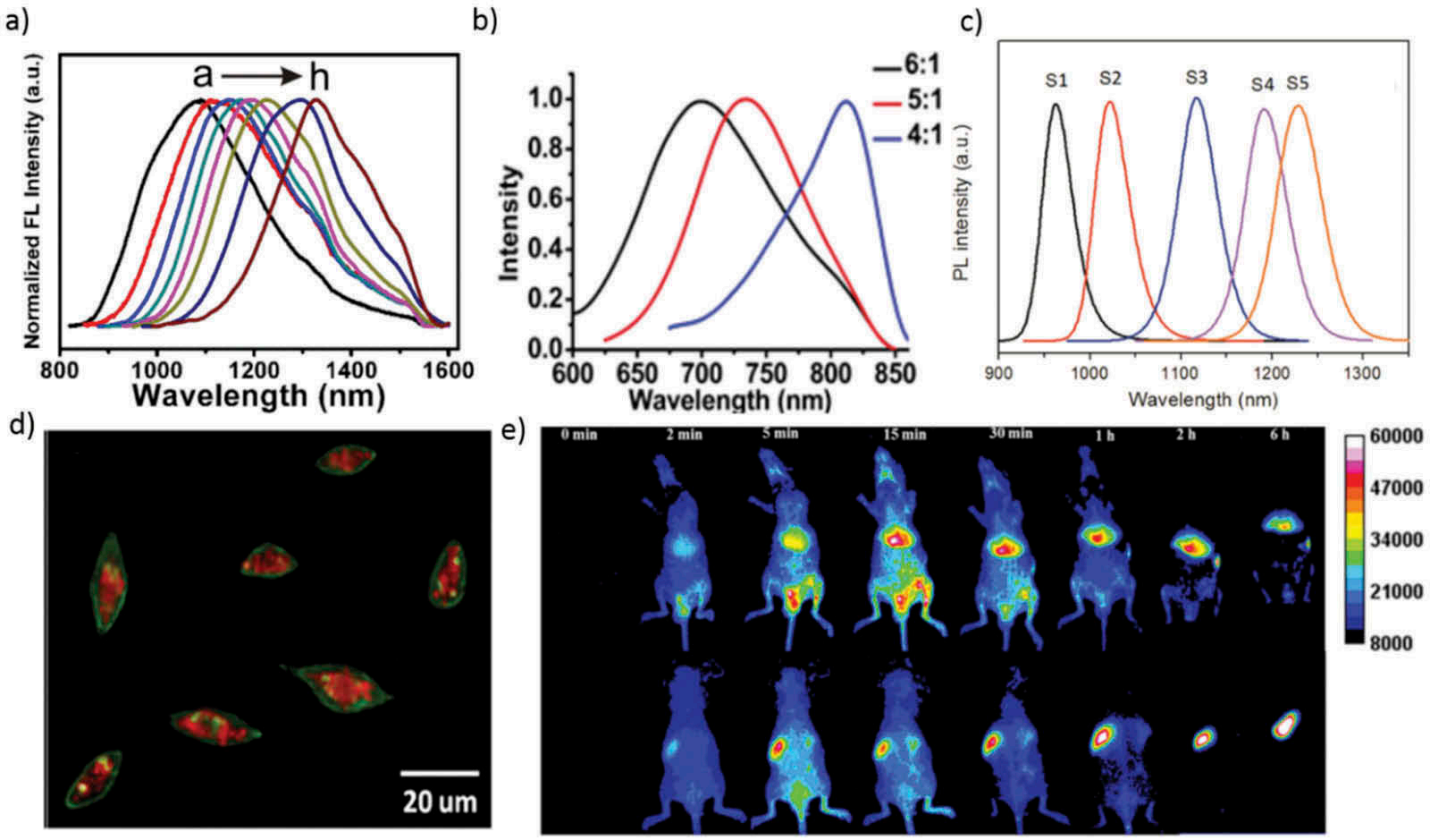
(a) PL spectra of Ag_2_Se QDs with different reaction times (b) PL spectra of monodispersed Ag_2_Se QDs with different sizes (molar ratio of Ag: Se = 6:1, 5:1, and 4:1). (c) Emission spectra of Ag_2_Se QDs capped with a multidentate polymer (samples S1−S5 corresponding to 0.5, 1, 1.5, 2, and 3 h reaction). (d) Overlay image (confocal laser scanning microscopy and NIR) of the Cu-2-{2-chloro-6-hydroxy-5-[(2-methyl-quinolin-8-ylamino)-methyl]-3-oxo-3H-xanthen-9-yl}-benzoic acid (CuFl) stained cells after incubation with the Ag_2_S-GSH-SNO NPs for 3 h. (e) *In vivo* imaging of PEGylated Ag_2_Se QDs in mice after intravenous injection. (Top row) Abdomen imaging; (bottom row) backside imaging. Reproduced with permission from (Figure 4(a), [52] Copyright 2013 The American Chemical Society; Figure 4(b), [60] Copyright 2012 The American Chemical Society; Figure 4(c), [54] Copyright 2014 The American Chemical Society; Figure 4(d), [53] Copyright 2013 The American Chemical Society; and Figure 4(e), [ 48] Copyright 2016 The American Chemical Society).

Gui et al. reported a facile synthesis of Ag_2_S QDs, which exhibit the size-dependent PL bands peaking in the NIR-II region, by cation exchange between visible-emitting CdS QDs and Ag^+^ ions in aqueous solution. Ag_2_S QDs produced minor effects on the aspects of altering cell proliferation and generating reactive oxygen species, indicating their ultralow cytotoxicity and excellent bio-compatibility [47]. Tang et al. reported the blood clearance, distribution, transformation, excretion, and toxicity of Ag_2_Se QDs modified with polyethylene glycol (PEG) in mice after intravenous injection. The PEGylated Ag_2_Se QDs prefers to accumulate in the spleen and liver but is almost transformed and cleared within 1 day. Remarkably, Ag released from the PEGylated Ag_2_Se QDs is excreted through both feces and urine, while the important element Se is barely excreted. Finally, the PEGylated Ag_2_Se QDs shows low toxicity, high performance, and proper bio-safety (Figure 4(e)) [48]. Hydrophilic Ag_2_S QDs hold great potential for *in vivo* imaging. Recently, Javidi et al. carried out cytotoxicity tests on human A549 and Hep G2 cell lines at concentrations of 6.25–200 µg/ml. The cytotoxicity test revealed that the QDs showed no substantial toxicity. *In vivo* imaging revealed that the Ag_2_S QDs penetrated the body of mice with excellent brightness. The water-soluble Ag_2_S QDs can be applied as NIR bio-imaging probes for diagnostics or *in vivo* imaging [49].

Song et al. demonstrated a method for preparing ternary AgInS_2_, quaternary AgZnInS, AgInS_2_/ZnS, and AgZnInS/ZnS nano compounds based on cation exchange. The photostability and *in vitro* experiment confirmed low cytotoxicity and extraordinarily promising applications in the arena of clinical diagnosis [50]. Ag_2_Te QDs and Ag_2_Te/ZnS core/shell QDs emitting in the NIR-II window (*λ*_em_ = 900−1300 nm) have been reported. These QDs possess small size (~7.6 nm) and are nontoxic to cells, which is perfect for optical bio-imaging [51]. Ag_2_Se QDs with tunable PL bands peaking in the NIR-II window (*λ*_em_ = 1000−1400 nm) have been produced by nucleation and growth using 1-octanethiol, a soft Lewis base, as a ligand (Figure 4(a)). The acquired Ag_2_Se QDs delivered tunable NIR-II fluorescence in the range from 1080 to 1330 nm. Ag_2_Se QDs had excellent fluorescence properties in water, which was essential for their potential applications in multicolor fluorescence imaging *in vivo* [52].

Tan et al. produced the NIR fluorescent Ag_2_S QDs in cultured Hep G2 cancer cells. QD precursors were delivered into the cells, where they reacted to produce QDs under the action of endogenous glutathione. By delivering QD precursors into cultured hepatoma carcinoma cell, Ag_2_S QDs with emission efficacy eligible for *in vivo* imaging were effectively synthesized with the aid of endogenous glutathione in the cells (Figure 4(d)) [53]. Later, Tan et al. synthesized Ag_2_Se QDs at room temperature with excellent PL QY in the NIR-II region (Figure 4(c)). The results break previous limits in synthetic conditions and provide a low-energy-consuming method for fabricating NIR-II QDs qualified for *in vivo* imaging [54,55]. Ge et al. thoroughly studied the bio-distribution and clearance in mice of NIR ultra-small Ag_2_Se QDs. The inductively coupled plasma mass spectrometry (ICP-MS) studies and bio-distribution fluorescent images of Ag_2_Se QDs in mouse organs confirmed that the Ag_2_Se QDs could be cleared rapidly from the mice body typically by renal excretion without accumulation. Pathological, biochemical analysis, and body weight results also showed that the Ag_2_Se QDs do not cause considerable toxicity in the *in vivo* environment [56].

Jiang et al. synthesized emission-tunable NIR Ag_2_S QDs with sizes ranging from below 1.5 to 4.6 nm. The PL spectra cover a range from 690 to 1227 nm, and it has excellent potential for the study of multiple nanodiagnostics and multicolor imaging *in vivo* environment [57]. Dong et al. synthesized Ag_2_Se QDs, through a facile solvothermal method with emission centered at 1300 nm. The *in vivo* experiment by administration of C_18_-PMH-PEG-Ag_2_Se QDs by intravenous tail injection attains imaging of organs and vascular structures down to ~100 μm [58]. Du et al. showed the combination of different coating ligands and solvents to attaining monodispersed Ag_2_S QDs [59]. Gu et al. successfully realize the synthesized water-dispersible Ag_2_Se QDs at 90 °C with less cytotoxic. The PL emission peak of Ag_2_Se QDs has been tuned from 700 to 730 nm and to 820 nm, corresponding to sizes from 1.5 ± 0.4 to 1.6 ± 0.4 nm and to 2.4 ± 0.5 nm with PL QYs more than 1% (Figure 4(b)). The NIR fluorescence of the Ag_2_Se QDs can penetrate through a living nude mouse [60].

Zhang et al. developed the bio-conjugation of Ag_2_S QDs with particular ligands, targeted labeling and imaging of different cell lines. The Ag_2_S QDs had negligible toxicity regarding cell proliferation, apoptosis and necrosis, ROS, and DNA damage. These NIR-II QDs had great potential for *in vivo* imaging, disease finding, and cancer diagnosis with clearance from the body [61]. Zhang et al. studied the long-term *in vivo* bio-distribution of Ag_2_S QDs functionalized with PEG and systematically examine the potential toxicity of Ag_2_S QDs overtime. The PEGylated-Ag_2_S QDs are accumulated in the liver and spleen after intravenous administration and can be slowly cleared. According to blood biochemistry, hematological analysis, and histological examinations, PEGylated-Ag_2_S QDs show slow toxicity when administrated to mice at doses of 15 to 30 mg/kg up to 2 months [62].

Qin et al. synthesized fluorescence-CT dual-mode nanoprobe by making use of DSPE-PEG2000-FA and other amphiphilic molecules to coat Ag_2_S QDs and iodinated oil simultaneously. *In vivo* experiments revealed that the probe has a rather long circulation time (blood half-life of 5.7 h), and the histopathological tissue tests indicated that it is not damaging to essential organs normal function [63]. Gui at al. demonstrated a facile aqueous synthesis of Ag_2_SQDs (2.6–3.7 nm) with bright and tunable PL emission in a broad range from the red to NIR-II (λ_em_ = 687 to 1096 nm), employing multidentate polymers as capping reagents [64]. Duman et al. established the aqueous synthesis of cationic, NIR-emitting Ag_2_S QDs (*λ*_em_ = 810–840 nm) with a mixed coating of MPA and Polyethylenimine (25 kDa) as new theranostic agent [65,66]. Shi et al. prepared high-quality, NIR-emitting Ag_2_Se QDs with distinct absorption features and high PL QYs using ODE–Se as the Se precursor [67]. Theodorou et al. demonstrated significant enhancement of PL intensity in the NIR-II region for Ag_2_S QDs, using Au nanostructures produced by colloidal lithography [68]. Zhang et al. effectively modified Ag_2_S QDs with a tumor-targeting small protein, affibody ZEGFR: 1907, via charge interaction. The resulting probe displays an EGFR targeted tumor imaging property, and it has a high potential for bio-imaging [69]. In 2014, Chen et al. prepared Ag_2_S QDs conjugated with TAT peptide in the second NIR window (NIR-II, 1.0–1.4 μm) for dynamically tracking of human mesenchymal stem cells *in vivo* with high sensitivity and high spatial and temporal resolution [70]. Recently, Chen et al. reviewed and discussed tracking the transplanted stem cells using fluorescent nanoprobes in the first and second biological window [71].

### 2.4. Group I-III-VI quantum dots

**Figure 5.**
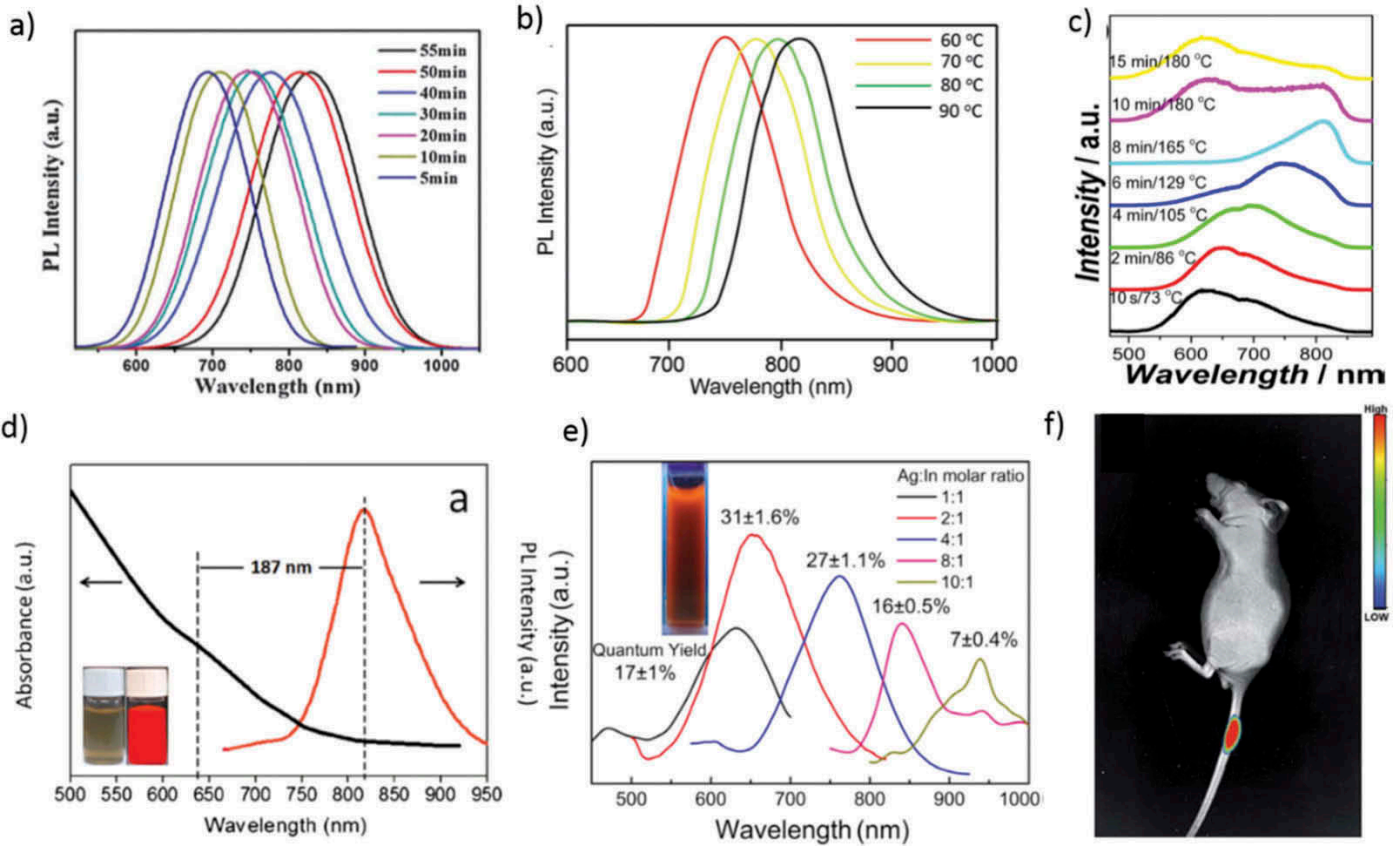
(a) The PL spectra of CuInS_2_ QDs at different reaction times. (b) PL spectra of RNase A-CuInS_2_ QDs at different reaction temperatures. (c) PL spectra of AgInS_2_/ZnS NCs by heating the Ag/In/Zn/S (1:1:0.5:2.5) solution from 90 to 180 °C. (d) Vis–NIR absorption and PL spectra of AgInS_2_QDs capped with a multidentate polymer synthesized for 105 min; (inset) bright-field image and PL image in pseudocolor. (e) PL spectra of AgInSe_2_ QDs prepared with different Ag: In molar ratios. The inset shows AgInSe_2_ QDs (Ag: In molar ratio of 2:1) in water, excited with a UV lamp at 365 nm. (f) Mice after treatment with the RNase A-CuInS_2_ QDs. Reproduced with permission from (Figure 5(a), [80] Copyright 2016 RSC Publishing; Figure 5(b), [74] Copyright 2017 RSC Publishing; Figure 5(c), [73] Copyright 2014 The American Chemical Society; Figure 5(d), [76] Copyright 2015 Elsevier publishing; Figure 5(e), [78] Copyright 2016 Elsevier publishing; and Figure 5(f), [74] Copyright 2017 RSC Publishing).

Recently, researchers concentrated on I−III−VI_2_ group semiconductor NPs of Cu or Ag-based materials, such as CuInS_2_, Cu(In,Ga)Se_2_, and AgInS_2_, due to their low toxicity [72]. Mao et al. synthesized the NIR-emitting AgInS_2_/ZnS QDs by heating an Ag/In/Zn/S solution and ending the reaction at 165 °C. Zinc concentration and rising temperature played significant roles in the formation of the NIR-emitting QDs (Figure 5(c)). Zinc enables the growth of the AgInS_2_/ZnS NCs at elevated temperatures without aggregation, and it suppresses the formation of defects which leads to an enhancement of the PL intensity of AgInS_2_/ZnS QDs. The beautiful NIR-emitting properties of the AgInS_2_/ZnS NCs make them potentially desirable for bio-imaging applications [73].

Xi et al. demonstrated a one-pot synthesis of water-soluble, NIR-emitting CuInS_2_ QDs using RNase A for *in vivo* imaging experiment; this result confirmed that they could be a good candidate for biomedical applications, particularly in the gastrointestinal system (Figure 5(b,f)) [74]. Jiang et al. synthesized Cu-doped Zn–In–S QDs by a hydrothermal method for labeling Hep G2 liver cancer cells. The cytotoxicity studies revealed that the viabilities of the cells incubated with increasing concentrations of CZIS/ZnS QDs all remained at a high level of more than 90%. Hence, the CZIS/ZnS NP is a promising material as the fluorescent probe for biological applications [75]. Tan et al. demonstrated water-dispersible AgInS_2_ QDs with bright NIR emission (Figure 5(d)). A type of multidentate polymer was synthesized and utilized as a compact capping ligand for the AgInS_2_ QDs. The multidentate polymer capping AgInS_2 _QDs are highly luminescent in the NIR window and have high photostability. Also, the QDs are stable in various media and have low cytotoxicity. Nude mice photoluminescence imaging shows that the multidentate polymer capping AgInS_2 _QDs can be well applied to *in vivo* imaging. These NIR fluorescent QDs have great potential for bio-medical applications [76].

Fahmi et al. prepared water-soluble AgInS_2_-ZnS QDs covered by oleylamine without surfactant or polymer for cancer cell staining. To demonstrate the targeting capability, folic acid was further conjugated with oleylamine encapsulated AgInS_2_-ZnS QDs for HeLa and MCF7 cancer cell staining [77]. Recently, a fast and straightforward synthesis of strongly NIR-luminescent AgInSe_2_–ZnSe QDs with tunable emissions in aqueous media has been reported. This method avoids high temperature and pressure and organic solvents to produce water-soluble AgInSe_2_–ZnSe QDs. The PL peaks of the AgInSe_2_–ZnSe QDs could be controlled in the ranging from 625 to 940 nm, along with PL QYs of 31% at maximum (Figure 5(e)). So, the AgInSe_2_–ZnSe QDs with high PL QY, emission tunability in the NIR range and low cytotoxicity are useful for cell labeling and bio-imaging [78]. Liu et al. reported highly water-soluble quality ternary CuInS_2_ QDs with MPA ligands as a stabilizer. They were used to label the liver cancer cells [79]. Qi et al. reported CuInS_2_ QDs with tunable emission from visible to NIR range (λ_em_ = 675–835 nm), the PL QYs of up to 8.7% were observed by just controlling the reaction time (Figure 5(a)) [80].

### 2.5. Group III-V quantum dots

**Figure 6.**
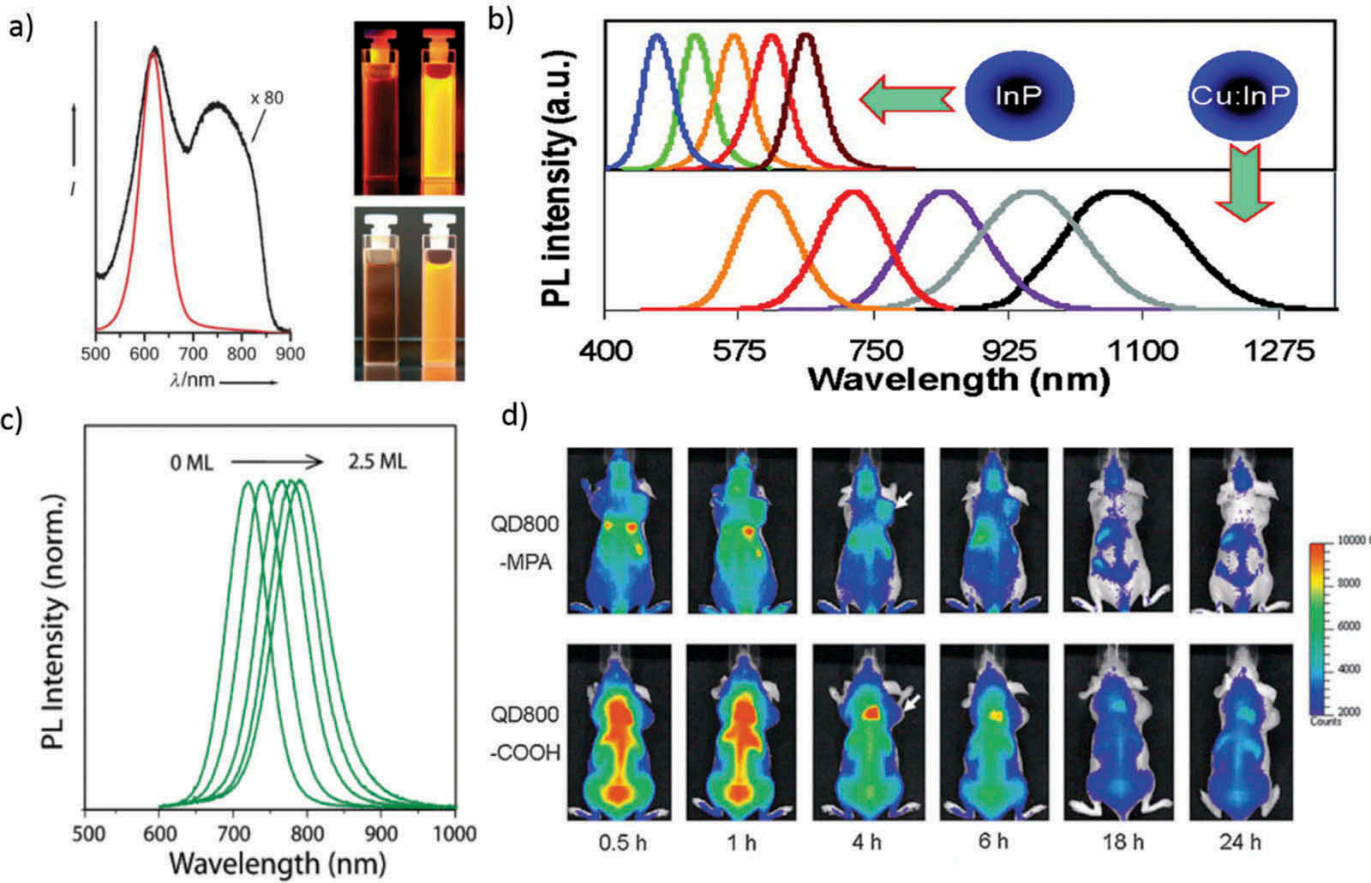
(a) PL spectra of InP QDs grown at 250 °C for 4 h before (black line) and after (red line) HF etching. Photographs of both colloidal solutions under UV illumination (inset top-366 nm) and UV/white light flash (inset bottom). (b) PL spectra of differently sized InP QDs and Cu: InP d-dots (c) PL of InAs (Zn_0.7_Cd_0.3_S) during shell growth from 0–2.5 monolayers. (d) *In vivo*, NIR fluorescence imaging of 22B tumor-bearing mice (arrows) injected with QD800-MPA and QD800-COOH, respectively. Reproduced with permission from (Figure 6(a), [83] Copyright 2008 Wiley Publishing; Figure 6(b), [81] Copyright 2014 The American Chemical Society; Figure 6(c), [82] Copyright 2010 The American Chemical Society; and Figure 6(d), [ 85] Copyright 2010 Wiley Publishing).

High-quality InAs QDs are likely the best candidate for NIR-light emitters (λ_em_ = 700–1400 nm) among II-VI, III–V, and IV-VI semiconductor QDs. InAs QDs with NIR-emitting properties were synthesized through a self-focusing of size distribution. One-pot synthesis can yield core-shell QDs with bright (up to 90% PL QY), stable, and narrow NIR-PL spectra, which is preferred for *in vivo* bio-medical imaging. Cu-doped InP QDs were successfully synthesized by epitaxial growth of a ZnSe diffusion barrier for the dopants. The Cu-dopant emission of the Cu: InP/ZnSe core/shell QDs covers a broad PL range from 630 to 1100 nm, and emission tunability is achieved by varying the size of the InP core (Figure 6(b)) [81].

Allen et al. prepared water-soluble InAs (ZnCdS) QDs with bright and stable PL bands peaking in the NIR region (Figure 6(c)). The NIR-emitting QDs can be functionalized to allow imaging of exact cellular proteins. Also, the efficacy of the NIR region for *in vivo* imaging is proved by the greater ability of InAs (ZnCdS) QDs to image tumor vasculature [82]. Colloidal III–V semiconductor QDs are well focused because of rich phenomena related to the effect of quantum confinement. However, studies of III–V QDs are limited due to synthetic complications. High-quality InP QDs has been adequately developed as luminescence probes for bio-imaging (Figure 6(a)) [83]. Bruns et al. introduced the short-wavelength infrared region (SWIR)-emitting InAs-based core-shell and core-shell–shell QDs as a flexible class of materials for functional biological imaging. These InAs-based QDs display higher QY and stability than previously described SWIR probes [84]. Gao et al. reported using 22B and LS174T tumor xenograft models, *in vivo* and ex vivo imaging studies show that QD800-MPA is hugely accumulated in the tumor part, which is essential for tumor detection in living mice (Figure 6(d)) [85].

### 2.6. Group IV quantum dots

It is a special kind of QDs based on only one element. Carbon dots (CDs) attracted much attention [86–91]. Zheng et al. reported NIR fluorescent Cy (cyanine dye) CD by the reaction of CyOH with PEG800 which is excellent photostability and bio-compatibility. CyCD gives strong absorption around 600 to 900 nm, NIR emission for *in vivo* imaging, favored uptake, and accumulation at tumors [92]. Pan et al. reported the CD fluorescent probes that are excited with NIR light to give a NIR emission. The red-emitting CD (*λ*_max_ = 683 nm) could be excited with an 850 nm light from a femtosecond pulsed laser. The NIR-emitting CDs exhibit an excellent water solubility, a narrow spectral emission, and high PL QY that is high as 16.8%. More remarkably, the NIR-emitting CDs have low cytotoxicity and are adapted for two-photon excitation. These CDs could not only be potentially employed for deep tissue two-photon excitation bio-imaging but also used as a carrier for the delivery of drugs that cannot enter into living cells directly [93]. Li et al. successfully prepared NIR-emitting CDs (R-CDs) using spinach as a precursor. The R-CDs showed an excellent water solubility, a remarkable photo-stability, an emission peak at 680 nm, a 15.34% PL QY, excellent compatibility, and low toxicity. R-CDs were proved to have excellent cellular labeling agent with lung cancer cells (A549) excited at 543 nm [94].

**Figure 7.**
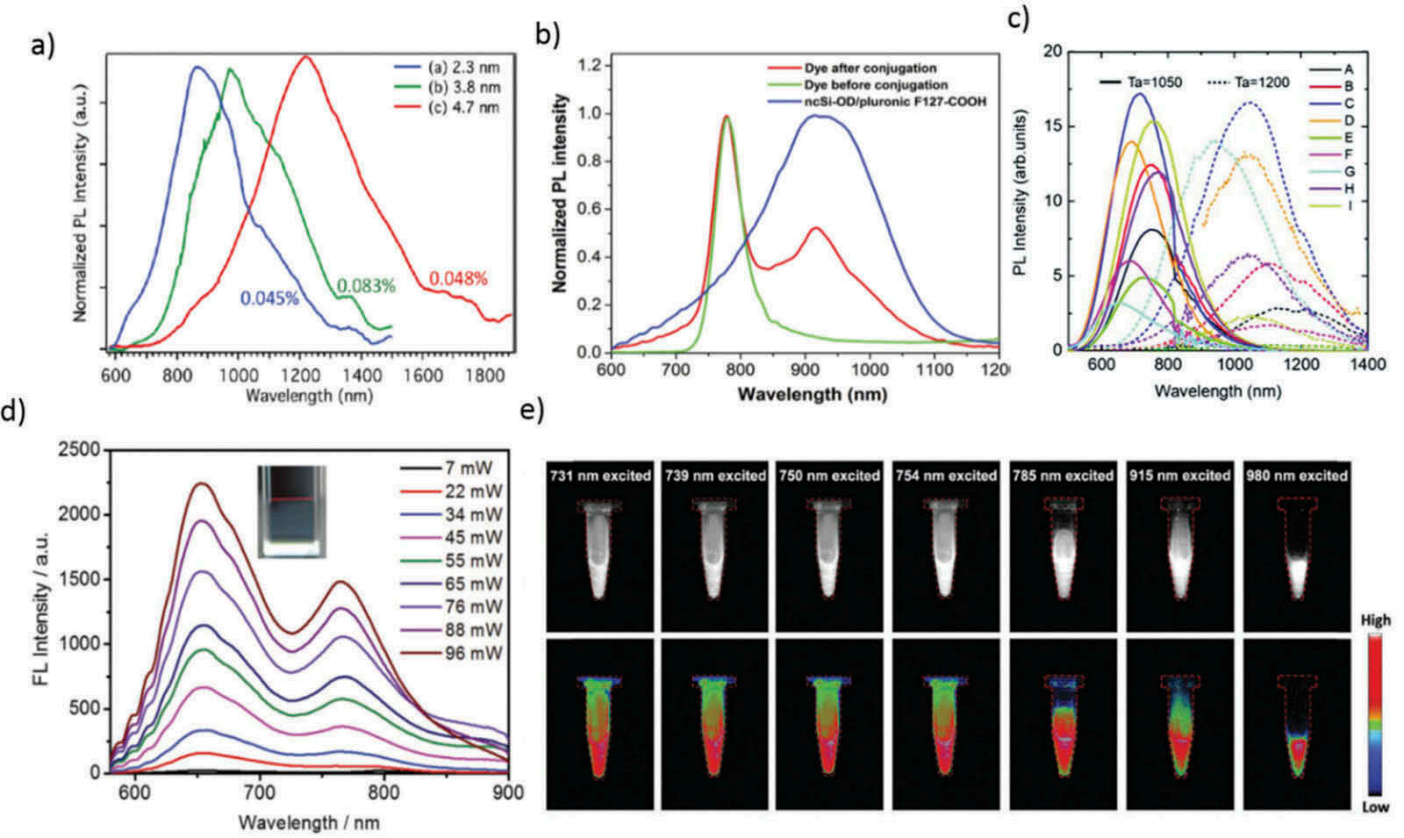
(a) PL spectra of tetrachloroethylene solutions of Ge-NC (100/0), Ge-NC (0/100), and Ge-NC (50/50) under 405 nm excitation. PL QY values are shown in percentage. (b) Normalized PL spectra of NCSi-OD/F127-COOH, HiLyte Fluor 750 amine dye and NCSi-OD/F127-COOH conjugated with the dye when excited at 350 nm. (c) PL spectra of a colloidal solution of codoped Si QDs. The growth temperatures are 1050 (solid) and 1200 °C (dashed). (d) Fluorescence spectra obtained for multiphoton excitation associated with incident intensities at; 1200 nm excitation (inset: excitation at 1200 nm and 22 mW). (e) SWIR fluorescence images of FCDs in aqueous dispersions, excited by lasers and captured with an InGaAs camera. Images in the bottom row are the heat maps of those in the upper row. Reproduced with permission from (Figure 7(a), [104] Copyright 2010 The American Chemical Society; Figure 7(b), [107] Copyright 2016 RSC Publishing; Figure 7(c), [141] Copyright 2016 RSC Publishing; Figure 7(d), [97] Copyright 2018 RSC Publishing; and Figure 7(e), [96] Copyright 2018 RSC Publishing).

Lan et al. prepared CDs codoped with S, Se via a hydrothermal method and NIR emissions of the CDs, with PL bands peaking at 731 and 820 nm and photothermal conversion efficiency of ~58.2%. The two-photon absorption cross-section (~30,045 GM; 1 GM = 10^−50^ cm^4^·s/photon) of the CDs are high enough to give a NIR emission. *In vitro* and *in vivo* tests suggested that CDs are useful phototheranostic agents for the two-photon excitation fluorescence imaging and photothermal therapy of cancer cells [95]. Li et al. showed that surface modified CDs could absorb the light for NIR emission. CDs in dimethyl sulfoxide (DMSO) exhibit an absorption band in the NIR-I region at 715 nm and NIR emission at 760 nm (PL QY reaching 10%), under NIR excitation. The NIR excitation and emission are both critical for CDs to realize the *in vivo* fluorescence imaging. Besides, two-photon and three-photon-induced fluorescence are observed in DMSO under excitation of light peaking in the NIR-II window. CDs modified with Poly(vinylpyrrolidone) (PVP) are used in the stomach of a living mouse for *in vivo* NIR fluorescence imaging. This is the first approach to get CDs with a NIR absorption band and NIR emissive CDs efficiently with either one-photon excitation in a NIR-I or multiphoton excitation in a NIR-II window for applications in NIR fluorescence imaging (Figure 7(d,e)) [96,97].

Group-IV QDs have recently attracted attention as a nontoxic alternative to classical II-VI and IV-VI QDs containing toxic elements such as Hg, Cd, and Pb. Bulk crystal of Ge is a typical indirect bandgap semiconductor with a narrow bandgap of 0.7 eV at room temperature and exhibits a poor optical performance because of its indirect bandgap character. However, the carrier confinement in an NP of Ge crystal with a diameter smaller than the bulk exciton Bohr radius (~11.6 nm) alters the energy structure. This situation allows for the overlapped wave functions of spatially confined carriers, leading to zero-phonon optical interband transitions for recombination as a result of the relaxation of the k-selection rule due to the Heisenberg uncertainty relation. After the 1993 report on yellow-emitting Ge QDs, it was found that control over size and structure of CDs results in emission tunability over the entire visible wavelength range [98–100].

In contrast, a few papers report the fluorescence colors in the NIR wavelength region [101–103]. Ruddy et al. synthesized, alkyl-terminated Ge QDs (2.3−11.3 nm) based on the reduction of GeI_4_/GeI_2_ mixtures. The NIR absorption (1.60−0.70 eV) and corresponding band-edge emission demonstrate the highly tunable quantum confinement effects in Ge QDs prepared using this mixed-valence precursor method (Figure 7(a)) [104]. Continuous tunability was achieved in a biological window of 900–1600 nm. However, the emission QY is still too low (~5%) for fluorescence biomarkers.

Bulk crystalline silicon (Si) is also included in a category of indirect bandgap semiconductors with a bandgap of 1.1 eV at room temperature. It is broadly known that the electronic structure in Si QD retains its indirect bandgap character. The effects of quantum confinement lead to the relaxation of the momentum conservation by the uncertainty principle (ΔχΔρ ≥ ħ/2), which allows for zero-phonon optical interband transitions. This theory predicts the appearance of PL peaks tailing on a low energy side. This tailing continues until ~1.1 eV, which is equal to the value of the bulk bandgap for crystalline Si. However, most of NIR PL spectra reported recently show homogeneous and Gaussian-like shape without long emission tails [105]. Although the PL QYs of the hydrogen-terminated Si QD are as low as ~5%, a merely substituting their surface hydrogen atoms with alkyl chains, which yields a covalent carbon-silicon linkage, increases the PL QYs up to ~70% [106].

Furthermore, the size-dependency of PL peak energy is achieved even after substitution of the surface ligands [107]. Presently, the high PL QY is postulated to arise from an increase in the radiative recombination rate [108], a dramatic reduction of the non-radiative channels [109,110], or a bandgap modulation from indirect to direct transitions [111]. Such an enhancement of PL QYs has been arguably observed for alkyl-terminated QDs with size-dependent PL bands and encourages the further development of QD-based optoelectronics devices [112–114].

Furthermore, Si QDs are nontoxic, easy availability, a long PL lifetime on μsec scale, along with brightness and it give fluorescence emission from UV to NIR wavelength range (Figure 7(b)) [115–124]. NIR-emitting Si QDs have been prepared by non-thermal Plasma or thermal disproportionation of hydrogen silsesquioxane (HSiO_1.5_), followed by hydrofluoric etching to liberate the Si QDs from the oxide matrix [125–127]. In most of the studies, HSiO_1.5_ purchased from Dow Corning (trade name FOx-17) are used for QD synthesis [128,129], but a similar compound could be derived from the hydrolysis of trichlorosilane or triethoxysilane [130,131]. Impurity doping of boron, phosphorus, or transition metals into diamond cubic Si lattice has also been achieved [132,133]. Sugimoto et al. developed a new approach for the formation of all-inorganic Si QDs that are codoped with boron and phosphorus with excellent stability in water without organic ligands, exhibit bright and stable luminescence in the NIR wavelength range [134]. Wang et al. reported for the first time the bio-medical use of Si QDs was as a fluorescence label to DNA [135]. Since then, Swihart and co-workers have significantly developed this research field [136]. As the cytotoxicity is strongly influenced by surface terminal groups [137,138], the amphiphilic molecules such as Pluronic F127 or PEG are frequently employed for encapsulating the core of Si QDs, yielding the high hydrophilicity and bio-compatibility. Si QDs exhibit a low absorption efficiency to the red-to-NIR lights. Therefore, it is difficult to attain the NIR-NIR excitation-emission bio-imaging in the single-photon excitation environment. To overcome this difficulty, He et al. reported possible compatibility of multiphoton excitation technique with Si QD [139]. Chandra et al. developed water-borne Si QD adapted for 2-photon excitation and used it to provide for the first time direct evidence of the NIR-NIR excitation-emission imaging [107]. Ravotto et al. presented a light-harvesting two-photon antenna using the emission of Si QDs. This result opens up the potential for bio-imaging applications, such as deep tissue imaging, high resolution, and low photodamage, coupled with the bright, long-lived, and oxygen-insensitive NIR luminescence of Si QDs [140]. Recently, Sakiyama et al. developed a long-lived luminescence of colloidal silicon QDs for time-gated fluorescence imaging in the second NIR window in biological tissue (Figure 7(c)) [141].

### 2.7. Sentinel lymph node mapping using NIR QDs

**Figure 8.**
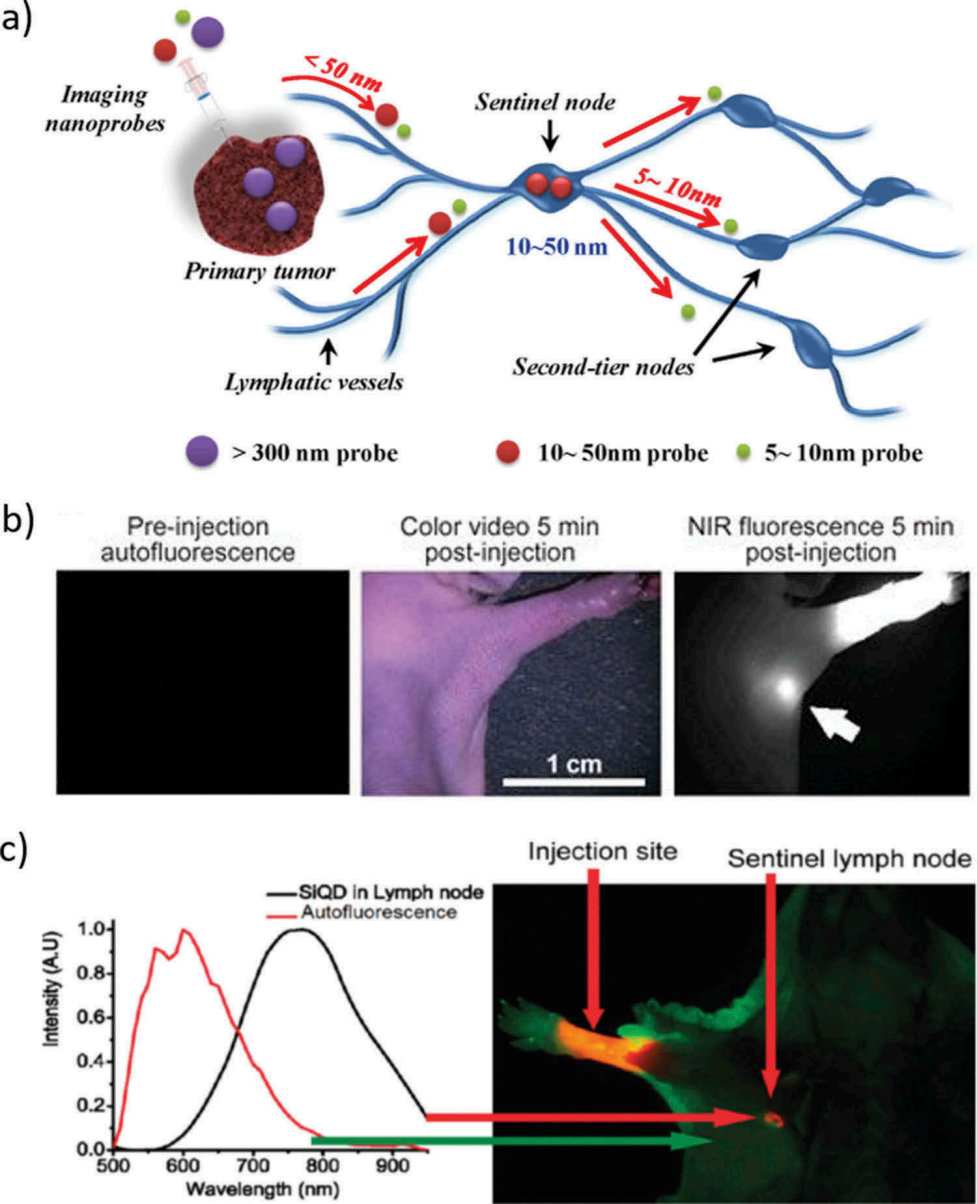
a). Schematic illustration of SLN mapping using a nanoscale imaging probe. Imaging probes on the 5–10 nm size scale can flow through the SLN into adjacent nodes in the chain; nanoprobes >300 nm in size rarely leave the injection site. Nanoprobes with a hydrodynamic diameter of 10–50 nm exhibit rapid uptake into SLN and do not leave. b). NIR QD sentinel lymph node mapping in the mouse; Images of mouse injected intradermally with 10 pmol of NIR QDs in the left paw. Left, pre-injection NIR autofluorescence image; middle, 5 min post-injection white light color video image; right, 5 min post-injection NIR fluorescence image. An arrow indicates the putative axillary sentinel lymph node. Fluorescence images have identical exposure times and normalization. c). Sentinel lymph node imaging following localization of Si QDs in an axillary position. Autofluorescence is coded in green, and the unmixed Si QD signal is coded in red. Reproduced with permission from (Figure 8(a), [143] Copyright 2012 The American Chemical Society; Figure 8(b), [145] Copyright 2004 Nature Publishing Group; and Figure 8(c), [150] Copyright 2011 The American Chemical Society).

Tumor metastasis usually occurs through the lymphatic or hematogenous pathway [142]. Therefore, the identification of metastasis within the sentinel lymph nodes (SLNs) is most important. Figure 8(a) outlines SLN mapping using nanoscale imaging probes with different sizes [143,144]. In 2004, Kim et al. reported type II NIR QDs with a polydentate phosphine coating for SLN mapping in both small and large animals. Injection of only 400 pmol of NIR QDs permits sentinel lymph nodes 1 cm deep to be imaged easily in real time using excitation intensity of only 5 mW/cm^2^ [145]. CdSe/ZnS core/shell QDs emitting around 655 nm was used for detection of axillary lymph node (ALN) as soon as 5 min and up to 24 h after the injection (Figure 8(b)). The maximum amount of QDs in the ALN was detected 60 min after the injection and corresponded to 2.42% of the injected dose. Most of the injected QDs remained at the injection site. No QDs were detected in other tissues, plasma, urine, and feces [146]. CdTe/CdS QDs are conjugating with R. reniformis luciferase (Luc8) used for *in vivo* SLN mapping because of their high sensitivity resulting from the limited background interference, as well as the high depth of tissue penetration of the luminescence [147]. Si et al. used cadmium tellurium QDs in rats for *in vivo* lymph node mapping. Both methylene blue and QD stained the sentinel lymph node quickly, but methylene blue was challenging to identify in the deep tissues and the LNs beyond the SLN. Furthermore, the blue-stained LNs remain dyed for only 2 h. In contrast, the QDs exhibited high target-to-background ratios in both the SLNs and the following LNs [148]. In 2010, Pons et al. used cadmium-free CuInS_2_/ZnS QDs for sentinel lymph node (LN) imaging with reduced toxicity. They used bright and photostable core/shell CuInS_2_/ZnS QDs with emission ranging from the red to NIR using air-stable compounds. After the water-soluble process, the QDs are used *in vivo* imaging of two regional LNs draining thoracic mammary fat pads in mice. The comparison of dose-dependent inflammatory response in the regional LNs induced by NIR CdTe-Se/CdZnS and CuInS_2_/ZnS functionalized with the same surface chemistry and show that CuInS_2_/ZnS presents a much-reduced toxicity compared to Cd-based QDs [149]. One year later, Erogbogbo et al. used bio-compatible silicon nanocrystals for sentinel lymph node mapping in live mice. When injected subcutaneously in the paw of a mouse, MSiQDs traveled through the lymphatics and migrated to an axillary location (Figure 8(c)) [150].

### 2.8. Advantages and disadvantages of NIR QDs

Cd-based QDs have been primarily used for bio-imaging based on their high quantum efficiencies. The high brightness and stability of PL of QDs are favorable for medical applications such as a fluorescence image-guided surgery for tumor removal. However, those QDs are made of CdSe and have cadmium selenide core with zinc sulfide shell (CdSe/ZnS), containing inherently toxic cadmium. Table 2 shows a summary of the toxicity studies of various types of QDs [136]. A variety of *in vivo* models are demonstrated for each assessment. As expected, QDs containing Cd and Pb show high toxicity in every case. High cytotoxicity of Ag_2_Se QD comes as a surprise although they show a good NIR PL performance based on the effect of quantum confinement. These advantages in PL performance offered by the QDs cannot outweigh the potential risk associated with the accumulation of their constituent elements in the human body.

**Table 2.** Different types of quantum dots used for *in vivo* toxicity studies in various animal models. Copyright 2013, Royal Society Of Chemistry. Reproduced with permission from Yong et al. [136].

Type of QD	Cells	Zebrafish	Xenopus embryo	Mouse	Rat	Macaque
CdSe	Toxic after oxidation, ~0.0625 mg ml^−1^	-	-	-	-	-
CdSe–ZnS^a^	Toxic after exposed to UV irradiation, ~ 1mg ml^−1^	LC_50_ values: 7–42 µM	Non-toxic at 4.2Χ10^9^ particles per cell	Non-toxic at 360 pmol- 6 nmol	Non-toxic at 15 nmol	-
CdSe–CdS–ZnS^b^	LC_50_: ~0.7 µM	-	-	Non-toxic at 125 nmol	Non-toxic at 3 nmol	Non-toxic at 25 mg kg^−1^
CdSe–CdS	LC_50_: 50–100 nM	-	-	Non-toxic at 48 pmol	-	-
CdTe	LC_50_: ~ 40 µg/mL	-	-	-	-	-
CdTe–ZnSe	Non-toxic at 1 mg ml^−1^	-	-	Non-toxic at 5 mg kg^−1^		
CdTe–ZnTe	Relatively Non-toxic at 150 µg ml^−1^	-	-	Non-toxic at 5 mg kg^−1^	-	-
CdTe–CdS–ZnS	Non-toxic at 3.0 µM	-	-	-	-	-
CdHgTe–ZnS	Adopted for cell imaging, no toxicity study was performed	-	-	-	-	-
CdSe–CdTe	Non-toxic at 1–10 µg ml^−1^ loaded in PLGA nanospheres	-	-	Sentinel lymph node mapping in mouse (dosage ~0.01 nmol) and pig (dosage ~0.4 nmol), no toxicity study was performed	-	-
CdTe_x_Se_1-x_ -CdS	Non-toxic at 200 µM	-	-	Nontoxic at 5 mg kg^−1^	-	-
InP–ZnS	Non-toxic at 100 mg ml^−1^	-	-	Intratumor administration of 74 µg for imaging, no toxicity study was performed	-	-
Mn:ZnSe	Non-toxic at 1.2 µM	-	-	-	-	-
ZnS:Mn–ZnS	Non-toxic at 400 µg ml^−1^	-	-	Non-toxic at 0.5 mg	-	-
Mn:CdSe_x_Te_1-x _-CdS	Non-toxic at 800 µg ml^−1^	-	-	Non-toxic at 10 mg kg^−1^	-	-
InAs_x_P_1-x_ – InP–ZnSe	-	-	-	Non-toxic at 150 pmol	-	-
InAs–ZnSe	-	-	-	Used for imaging, no toxicity study was performed	Sentinel lymph node imaging, no toxicity study was performed	-
InAs–InP–ZnSe	LC_50_: ~100 nmol L^−1^	-	-	Non-toxic at 0.5 nmol	-	-
CuInSe_2_	-	-	-	Sentinel lymph node imaging, no toxicity study was performed	-	-
CuInS_2_	LC_50_: ~300 mg ml^−1^	-	-	Non-toxic at 0.1–0.2 µmol (based on Cu atoms), or 0.5 mg	-	-
Ag_2_Se	Non-toxic at 47.4 µg ml^−1^	-	-	Non-toxic at 0.15 mg	-	-
PbSe	Non-toxic at 100 µg ml^−1^ SiO_2_ coated	-	-	-	-	-
PbS	Non-toxic at 220 µg ml^−1^	Caused Malformations above 40 µg ml^−1^	-	Non-toxic at 25 mg kg^−1^ or 0.3 mg	-	-
ZnS	Non-toxic at 100 µM	-	-	-	-	-
Si	Non-toxic at 0.2 mg ml^−1^	-	-	Non-toxic at 380 mg kg^−1^ or 60 nmol	-	-
CdSe–CdS Quantum rods	LC_50_: less than 500 µM	-	-	-	-	-
CdSe–CdS–ZnS Quantum rods	Non-toxic at 500 µM and 2.8 µg ml^−1^	-	-	Non-toxic at 1 mg	-	-

^a^Core–shell structure. ^b^Core–shell–shell structure

In Table 2, PbSe are non-toxic until 100 μg/mL. It can be predicted that PbS also show a similar assessment of toxicity. PbS/SiO_2_ core-shell QDs are non-toxic until 220 μg/mL in cell culture due to the presence of the nontoxic shell, and this amount is as high as that of Si QD. It is noted that the amount for the availability of the PbS/SiO_2_ QD is very less for nonhuman primate study of QD toxicity. However, Si QD demonstrates the non-toxicity even at 380 mg/kg which is 15 times larger than the limitation of the PbS/SiO_2_ QD (~25 mg/kg). This comparison suggests that covering the toxic cores with less-toxic materials could not be the answer to solve the problems. In addition, the presence of heavy-metals in these QDs makes them a potential risk to the environment as their use increases and products reach the end of life. Pb-based QDs also emit high-intensity light up to second near infra-red region; researchers were used in lower concentration for bio-imaging after surface modification.

Many papers report that Ag-based QDs are a non-toxic probe for cells which is emitted light in the second infrared region. Ag_2_S QDs may be better than Cd, Pb, and Hg although more careful assessment is necessary. CuInS_2_ and AgInS_2_ QDs are much more bio-compatible than toxic QDs like HgTe, CdHgTe, CdTeSe, CdTeSe/CdS, PbS, and Au/PbS QDs. InAs-based core-shell QDs does not show high cytotoxicity and rapidly eliminated through renal clearance which reduces potential toxicity to the body. Si and Ge QDs are non-toxic to the human body, and it will emit the light in the near infra-red region. For Si QDs, the drawback is low photoabsorption coefficient because of their energy structure that retains indirect bandgap nature observed for bulky crystal. Surface chemistry is allowing the efficient fluorescence resonance energy transfer between surface ligands working as absorbers and Si QDs is expected to solve this problem. For Ge NCs, the PL QY should be increased.

## 3. Summary and perspectives

In this review, we summarized recent progress in bio-markers of NIR-emitting QDs for fluorescence bio-imaging. The review focuses on the tremendous efforts invested in the colloidal synthesis and functionalization of the water-borne NIR-emitting-QDs with different chemical compositions. Especially, we outlined the applications of NIR-QDs in the area of *in vitro* and *in vivo* imaging, using their unique optical properties, low toxicity, surface functionalization, high photostability and brightness. Besides, we summarized the *in vivo* fluorescence imaging of sentinel lymph node and tumors. Thanks for the variety of constantly evolving synthetic strategy for colloidal QDs and their improved optical performance, there are many possible QDs at present, which exhibit high stability in an aqueous buffer solution, a broad wavelength tunability in the biological window, and a significantly improved PL QY. Recent studies for the NIR-emitting QDs of IV, I-VI, II-VI, IV-VI, III-V, and I-III-VI semiconductors are picked up to be demonstrated herein. By controlling the size of their QDs, the accessible emission wavelength for bio-imaging is extended to the NIR-I and NIR-II window. Furthermore, the QDs shown have a unique optical performance such as a large multiphoton absorbance cross-section, yielding a multiphoton absorption of NIR light to result in the successful multiphoton imaging and an upconverted emission imaging. According to this scenario, synthesis and optical performance of NIR-emitting Cd-, Pb and Hg-based QDs are also described although their toxicities are known well. According to the scenario demonstrated in Table 2 reports the encapsulation of these toxic QDs with SiO_2_ or polymers, but the covering the toxic cores with less-toxic materials could not be the answer to solve the cytotoxic problems. QDs with diameters larger than dye-molecules enables chemists to access to the photoacoustic imaging due to larger vibration of their crystalline lattice structures. Meanwhile, IV QDs such as C, Si, and Ge provide advantages in terms of ultralow cytotoxicity, bio-compatibility and the ability to form a covalent linkage with organic ligands, resulting in high stability even in the aqueous buffer containing bovine serum albumin. In particular, Si is an abundant element and takes advantage of its high QY greater than 60%. As QDs of Si and Ge provide a long PL lifetime on μsec scale, biochemists can avoid the unwanted effect of auto-fluorescence from cell molecules on bio-imaging, and meet a good opportunity to be accessible to time-gated bio-imaging. Moreover, the low toxicity of Si and Ge QDs has relived some of the concerns associated with their use *in vivo* environment. Further investigations of the toxicological impacts and bio-friendly surface chemistry in different biological models are needed, but these distinguishing features observed in the NIR-emitting QDs can expand their use to various pathways for beautiful imaging that includes photothermal cellular imaging under *in vitro* and *in vivo* environment, leading to the next generation concept of nanomedicine ‘Theranostics’ that is a combination of diagnosis and therapy with medical treatments. In the future, we would predict a powerful technique using NIR-QDs for fluorescence *in vivo* imaging in terms of high sensitivity and deep tissue penetration capacity. Alternatively, the synthesis of biocompatible, stable NIR-QDs using green approach is mandated. Also, we need effective synthetic methods to get high quantum efficiency NIR QDs with a narrow full-width half maximum.
